# Supplementary material for: Telecom-wavelength quantum teleportation using frequency-converted photons from remote quantum dots
Source: Nat Commun. 2025 Nov 17;16:10027. doi: 10.1038/s41467-025-65912-8 (PMC12623975; doi:10.1038/s41467-025-65912-8)
Supplement: Supplementary file 1 — Supplementary Information [file 41467_2025_65912_MOESM1_ESM.pdf]

Supplementary Material for
"Telecom-Wavelength Quantum Teleportation using Frequency-Converted Photons from Remote Quantum Dots"

Tim Strobel,[1,*] Michal Vyvlecka,[1] Ilenia Neureuther,[1] Tobias Bauer,[2] Marlon Schäfer,[2] Stefan Kazmaier,[1] Nand Lal Sharma,[3] Raphael Joos,[1] Jonas H. Weber,[1] Cornelius Nawrath,[1] Weijie Nie,[3] Ghata Bhayani,[3] Caspar Hopfmann,[3] Christoph Becher,[2] Peter Michler,[1] and Simone Luca Portalupi[1]

[1]*Institut für Halbleiteroptik und Funktionelle Grenzflächen, Center for Integrated Quantum Science and Technology (IQST) and SCoPE, University of Stuttgart, Allmandring 3, 70569 Stuttgart, Germany*
[2]*Fachrichtung Physik, Universität des Saarlandes, Campus E2.6, 66123 Saarbrücken, Germany*
[3]*Institute for Integrative Nanosciences, Leibniz IFW Dresden, Helmholtzstraße 20, 01069 Dresden, Germany*

This supplementary document provides more in-depth studies about the utilized quantum dot sources. Furthermore, the data evaluation process and teleportation measurement results in further Bell state projections are given. Finally, the theoretical model employed to describe the teleportation experiment is presented.

Supplementary Note 1. SOURCE CHARACTERIZATION

In the following, the optical and quantum-optical properties of the utilized QDs are given.

A. Excitation

For pulsed two-photon excitation of both QDs, we employed a Coherent Mira Ti:sapphire laser, generating pulses of approximately 3 ps duration at a repetition rate of 76.2 MHz. The pulses were temporally broadened to approximately 23 ps using a 4f pulse-shaping setup, resulting in a temporal and spectral profile well approximated by a Gaussian. To increase the repetition rate, the shaped pulses were subsequently passed through cascaded unbalanced Mach–Zehnder interferometers, effectively quadrupling the rate to 304.8 MHz. As the pulses pass through different fiber paths in the unbalanced Mach-Zehnder interferometers, the polarization of each of the respective four repetitions is slightly different. This leads to different side-peak heights in the correlations. The optimal TPE conditions are achieved by adding a fraction of continuous-wave above-barrier pumping (a magnitude below saturation) with laser diodes to stabilize the charge environment of the QD. For QD1, the average resonant laser power was 8.6 µW with 2.1 µW of above-barrier pumping at 635 nm. QD2 was excited with a resonant power of 20.3 µW with 0.56 µW above-barrier pumping at 532 nm. We want to note here that the actual power required to reach the π-pulse for QD2 is 4 µW. As both QDs are excited by the same resonant laser, we used the collimation of the excitation beam before impinging the microscope objective as a tuning nob for the effective excitation power, leading to a higher measured power of 20.3 µW in the actual experiment. Finally, the excitation lasers are focused onto the sample with a Zeiss LD EC Epiplan-Neofluar 100x/0.75 DIC M27 objective.

B. Emission spectrum

The emission spectrum of QD1 is shown in Supplementary Figure 1a. The resulting above-band excitation spectrum is given in green. The filtered XX emission spectrum is given in blue when exciting the QD in pulsed two-photon excitation (TPE) at the π-pulse. The XX binding energy of QD1 is 3.71 meV. The X and XX emission spectrum of QD2 excited in pulsed two-photon excitation at the π-pulse is shown in Supplementary Figure 2a. The excitation laser is well suppressed, and no residual QD emission lines are coupled. The XX binding energy of QD2 is 3.81 meV. The X and XX emission lines were identified via fine-structure splitting (FSS) measurements (see Supplementary Note 1 E). In the respective FSS measurement, the oscillation of the X and XX lines will be 90° out of phase (compare blue and yellow data in Supplementary Figure 3.

* t.strobel@ihfg.uni-stuttgart.de

C. Spectral diffusion mechanisms

In the presented type of QD structures, the usual sources of inhomogeneous broadening are charge, spin noise, and phonon coupling [1, 2]. To clearly identify the underlying broadening mechanisms at hand with certainty, more dedicated measurements must be performed (see [3]). The QDs presented in this work are blinking, show bright trion lines in pure above-barrier excitation, and require a fractional amount of continuous-wave above-barrier excitation in addition to the pulsed resonant laser for efficient TPE. Together, the latter three observations are a strong indication of additional charge carriers being present in the vicinity of the QDs. We therefore assume that charge noise plays a major role in the spectral broadening of the QD emission.

D. Rabi oscillations

Proper preparation of the three-level system is confirmed by Rabi oscillations shown in Supplementary Figure 1b for QD1 and Supplementary Figure 2b for QD2. Both X and XX of QD2 oscillate in phase, with increasing excitation power.

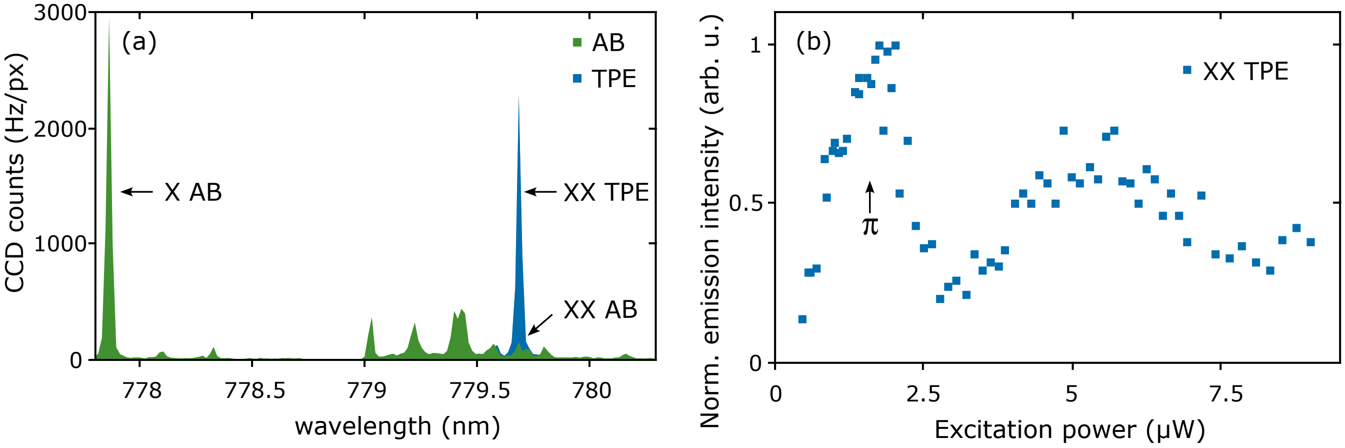

Supplementary Figure 1. **Emission spectrum and Rabi oscillations of QD1.** **(a)** Two emission spectra of QD1 are shown. In green, the QD is pumped with above-band (AB) excitation, while in blue, the QD is excited in two-photon excitation (TPE) at the π-pulse, and the XX line is spectrally filtered. For the TPE, the laser is filtered out with two volume Bragg gratings, and the XX emission line is coupled into a single-mode fiber using a volume Bragg grating and sent onto a spectrometer. **(b)** Laser-pulse-power-dependent Rabi oscillations for the filtered XX emission at an excitation rate of 76.2 MHz are shown.

E. Fine-structure splitting

The fine-structure splitting (FSS) of the QDs is determined via a rotating waveplate measurement. The QD emission is transmitted through a half-waveplate followed by a polarizer. The QD emission is monitored on the spectrometer while the waveplate angle is changed. The resulting energy of the emission line is plotted against the polarization angle θ as shown for QD1 and QD2 in Supplementary Figure 3. The data are fit with the following model:

$$f(\theta) = a \cdot \sin(b \cdot \theta + c) + d, \tag{1}$$

with amplitude a, frequency b, phase c and offset d, where the FSS corresponds to double the amplitude $\delta_i = 2a$. For the X emission of QD1 the fit yields $\delta_{1,X} = 10.4(2)\,\mu\text{eV}$ and for QD2 the values are $\delta_{2,\text{X}} = 2.5(4)\,\mu\text{eV}$ and $\delta_{2,\text{XX}} = 1.7(5)\,\mu\text{eV}$. The latter two result in an average FSS of $\delta_2 = 2.1(3)\,\mu\text{eV}$ for QD2.

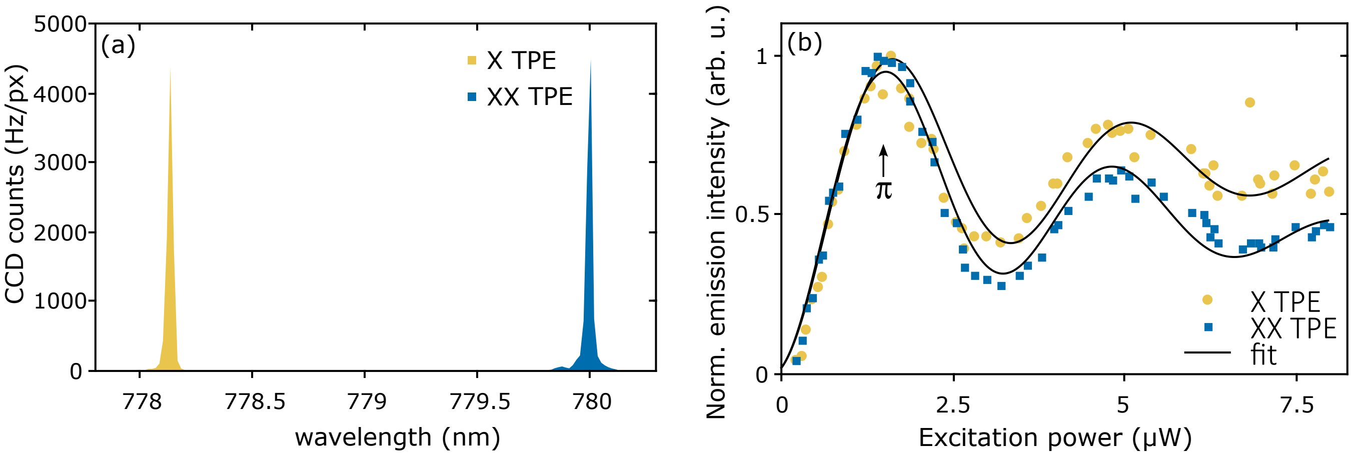

Supplementary Figure 2. **Emission spectrum and Rabi oscillations of QD2 in two-photon excitation (TPE).** **(a)** QD2 is excited in pulsed TPE at the π-pulse. The laser is filtered out with two volume Bragg gratings. X and XX emission lines are coupled into separate fibers using volume Bragg gratings and sent onto a spectrometer. **(b)** Laser-pulse-power-dependent Rabi oscillations at an excitation rate of 76.2 MHz for the filtered X and XX emissions are shown. The normalized data are offset to improve clarity. A fit to the data is shown in black.

F. Probability of multiphoton events

The second-order correlation function, $g^{(2)}(\tau)$, of all three photons involved in the teleportation protocol was measured using a Hanbury Brown-Twiss setup. The quantum dots were excited by a pulsed laser with a repetition rate $\nu = 304.8\,\text{MHz}$. The second-order correlation function of Photon 3, $g_3^{(2)}(\tau)$, was measured using SNSPD detectors with an efficiency $\eta = 85\,\%$ at 780 nm and a time resolution 44 ps (full width at half maximum). The second-order correlation functions $g_1^{(2)}(\tau)$ and $g_2^{(2)}(\tau)$, corresponding to Photon 1 and Photon 2, respectively, were measured after quantum frequency conversion of the photons by SNSPD detectors with an efficiency $\eta = 85\,\%$ at 1550 nm and a time resolution 37 ps. The measured second-order correlation functions for individual photons are shown in Supplementary Figure 4a-c. The bunching of the data around zero time delay is caused by blinking, a phenomenon where the QD emission is temporarily quenched. The bunching of QD1 is described by a mono-exponential decay with a decay constant of 10 ns and an optically active fraction of 0.26. The blinking of QD2 can be described by a biexponential decay on timescales of 12 ns and 34 ns, yielding an optically active fraction of 0.43. The temporal characteristics of the blinking are consistent with charge noise, allowing us to attribute its origin to fluctuations in the local charge environment.
The pronounced differences in peak maxima observed in panel b) arise from a slight misalignment of the cascaded Mach-Zehnder interferometers used for rate amplification. When light traverses different optical fiber paths within the interferometers, it experiences varying degrees of birefringence. As a result, the polarization of individual pulses can deviate from alignment with the QD axis. Since excitation efficiency depends on polarization alignment, misaligned pulses excite the QD less effectively. This leads to reduced emission intensity for those pulses, causing the observed imbalance in the heights of adjacent peaks in the cross-correlation measurement.

The values of the individual second-order correlation functions at zero time delay, $\tau = 0$, as a function of the temporal filtering window size $\Delta\tau$, are shown in Supplementary Figure 4d-f. The values of $g_{i,\Delta\tau}^{(2)}(0)$ were calculated numerically by integrating over all detected photons without background subtraction; the area of the central peak was compared to the area of side peaks at the Poissonian level to account for blinking effect. The single-photon purity of Photon 1 and Photon 2 is reduced, resulting in higher values of the corresponding second-order correlation functions due to CW noise from the frequency conversion process.

To determine the number of unwanted three-fold coincidences, the two-photon generation probabilities have to be calculated from the dependences of individual $g^{(2)}(0)$ on the time window size. The two-photon component was estimated under an assumption that multi-photon emission consists only of a two-photon component, $p_{\geq 3} = 0$, as [4]

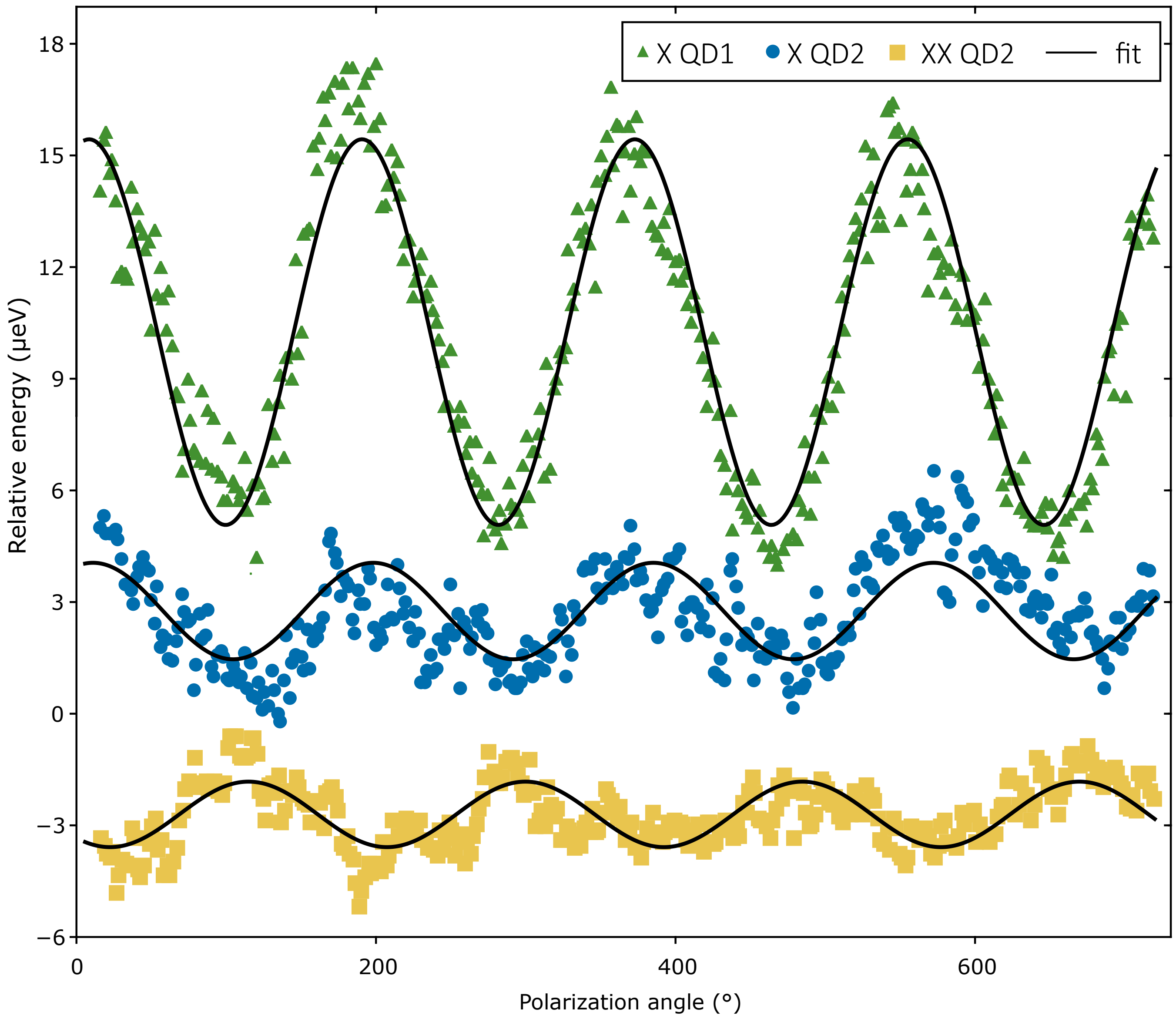

Supplementary Figure 3. **Fine-structure splitting (FSS) of the employed quantum dot** The given data points the FSS of QD1 (QD2) obtained via a rotating waveplate measurement. The X emission of QD1 is shown in green, while the X (XX) emission of QD2 is shown in blue (yellow). A $\sin\theta$ fit to the data is shown in black.

$$p_2^i(\Delta\tau) \leq \frac{1 - Bg^{(2)}_{i,\Delta\tau}(0) - \sqrt{1 - 2Bg^{(2)}_{i,\Delta\tau}(0)}}{g^{(2)}_{i,\Delta\tau}(0)}\,, \tag{2}$$

where B is the quantum dot single-photon count rate at the detector (measured single-photon counts from the QD on the detector) and index i is marking the corresponding photon, and $\Delta\tau$ is reminding that the second-order correlation function depends on the used time window size. Then all possible combinations of detector clicks leading to unwanted three-fold coincidences are given as

$$CC^3_{\mathrm{unwanted}}(\Delta\tau) = \frac{1}{2}p_2^2(\Delta\tau)p_1^3(\Delta\tau) + \frac{1}{2}p_2^1(\Delta\tau)p_1^3(\Delta\tau) + \frac{1}{2}p_1^1(\Delta\tau)p_1^2(\Delta\tau)p_2^3(\Delta\tau)$$
$$+\frac{1}{4}p_2^2(\Delta\tau)p_2^3(\Delta\tau) + \frac{1}{4}p_2^1(\Delta\tau)p_2^3(\Delta\tau) + \frac{1}{8}p_2^1(\Delta\tau)p_2^2(\Delta\tau)p_2^3(\Delta\tau)\ , \tag{3}$$

where the lower index marks the probabilities of single and two-photon generation and the upper index distinguishes between different photons involved in the teleportation experiment. The number of wanted three-fold coincidences can be given as

$$CC^3_{\mathrm{wanted}}(\Delta\tau) = p_1^1(\Delta\tau)p_1^2(\Delta\tau)p_1^3(\Delta\tau) \tag{4}$$

Now the ratio of wanted to all three-fold coincidence, the so-called k-factor, is defined as

$$k(\Delta\tau) = \frac{CC^3_{\mathrm{wanted}}(\Delta\tau)}{CC^3_{\mathrm{wanted}}(\Delta\tau) + CC^3_{\mathrm{unwanted}}(\Delta\tau)} \tag{5}$$

The k-factor obtained from experimentally measured $g^{(2)}_{i,\Delta\tau}(0)$ is shown by Supplementary Figure 4d. The k-factor for a time window of 70 ps is given as $k(70\,\mathrm{ps}) = 0.85$.

G. Entanglement and fine-structure splitting

Ideally, QD2 emits a maximally entangled two-photon state $|\psi(t)\rangle$ with a phase given by the QD fine-structure splitting (FSS) δ_2 [5–7]:

$$|\psi(t)\rangle = \frac{1}{\sqrt{2}}\left(|H_{XX}H_X\rangle + \exp\left(i\,\delta_2\,t/\hbar\right)|V_{XX}V_X\rangle\right), \tag{6}$$

where $|H_{XX}H_X\rangle$ ($|V_{XX}V_X\rangle$) corresponds to the state where X and XX are both horizontally (vertically) polarized. Further $\hbar$ is the reduced Planck constant and t the time between XX and X emission. This FSS-induced oscillation translates into a rotation in the outer off-diagonals of the two-photon density matrix $\rho^{\mathrm{XX,X}}_{VV,HH/HH,VV}$:

$$\rho^{\mathrm{XX,X}} = \frac{1}{2}\cdot\begin{pmatrix} 1 & 0 & 0 & e^{-i\frac{\delta_2}{\hbar}t} \\ 0 & 0 & 0 & 0 \\ 0 & 0 & 0 & 0 \\ e^{+i\frac{\delta_2}{\hbar}t} & 0 & 0 & 1 \end{pmatrix}. \tag{7}$$

For more details, see [8], where QD2 was investigated thoroughly. Supplementary Figure 5 depicts the two-photon density matrix before (a,b) and after (c,d) frequency conversion for a 8 ps integration window. The QFC process preserves the entangled state with fidelities to $|H_{XX}H_X\rangle + |V_{XX}V_X\rangle$ of $F_{\mathrm{init}} = 0.974$ before and $F_{\mathrm{conv}} = 0.972$ after conversion for the 8 ps time window. In the pre-conversion measurement, both photons were projected before any birefringent elements. Post-QFC, the XX photon traversed several meters of birefringent fiber before projection, introducing a polarization phase shift θ when not compensated. This rotates the coherence terms $\rho^{\mathrm{XX,X}}_{14}$ and $\rho^{\mathrm{XX,X}}_{41}$ as:

$$\rho^{\mathrm{XX,X}} = \frac{1}{2}\begin{pmatrix} 1 & 0 & 0 & (\cos(2\theta) - i\sin(2\theta))e^{-i\frac{\delta_2}{\hbar}t} \\ 0 & 0 & 0 & 0 \\ 0 & 0 & 0 & 0 \\ (\cos(2\theta) + i\sin(2\theta))e^{+i\frac{\delta_2}{\hbar}t} & 0 & 0 & 1 \end{pmatrix}. \tag{8}$$

For small θ, the imaginary part changes linearly, while the real part remains nearly constant, as seen in Supplementary Figure 5. The QFC data were acquired over 15.13 h without active polarization stabilization; fiber birefringence was compensated only at the start. We attribute the coherence variation to polarization drift in the fiber. When aiming for longer fiber lengths, especially in deployed experiments, the described polarization fluctuations become critical and require a more delicate compensation routine. Fast single-photon detectors also allow for resolving the investigated state's temporal evolution. Supplementary Figure 6a, b depicts the oscillatory behavior described in Supplementary Equation 7 before and after QFC.

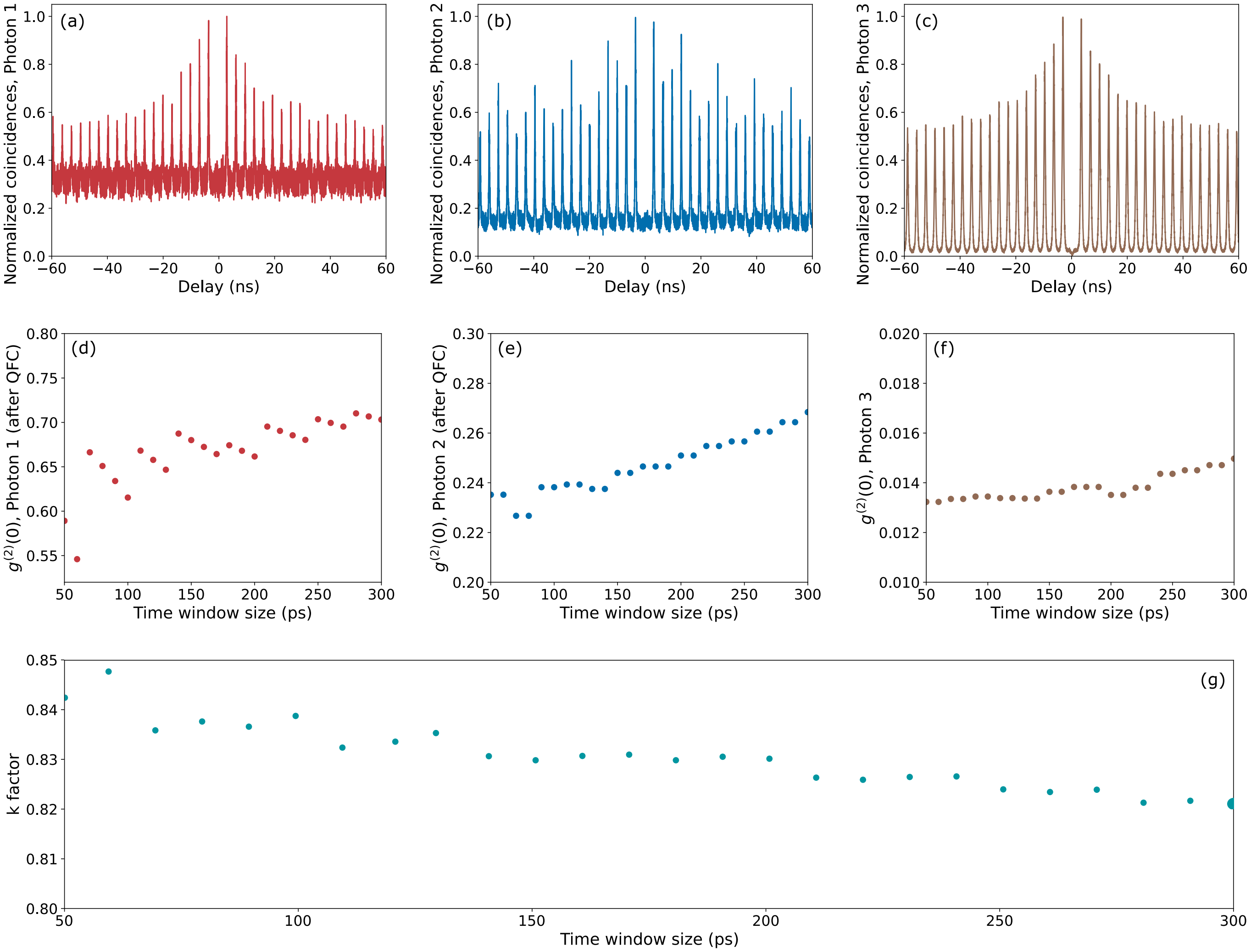

Supplementary Figure 4. **Experimentally measured second-order correlation functions $g^{(2)}(\tau)$ for individual photons and corresponding k-factor as a function of the temporal filtering window size.**
(a) Second-order correlation functions for Photon 1 after quantum frequency conversion. **(b)** Second-order correlation functions for Photon 2 after quantum frequency conversion. **(c)** Second-order correlation functions for Photon 3. **(d)** Second-order correlation functions for Photon 1 after quantum frequency conversion at zero time delay. **(e)** Second-order correlation functions for Photon 2 after quantum frequency conversion at zero time delay. **(f)** Second-order correlation functions for Photon 3 at zero time delay. **(d)** Corresponding k-factor calculated from experimentally measured second-order correlation functions, calculated as defined by Supplementary Equation 5.

H. Two-photon interference histogram

Supplementary Figure 7 depicts the histogram of a two-photon-interference measurement between QD1 and QD2 after QFC. The data were recorded in a parallel polarization setting. A bunching of side peaks, near to zero time delay, is due to the blinking of the sources on a time scale of 10 ns. Cascaded Mach-Zehnder interferometers are used for the excitation rate amplification (allowing to go from an excitation rate of 76.2 MHz to 304.8 MHz). A small imbalance among the interferometers leads to the observed imbalance in the height of neighboring peaks. In the correlation histogram, the observed CW background originates from the QFC noise. The data are normalized by fitting the peaks at the Poissonian level (side peaks beyond any blinking time scale) with Lorentzian functions and averaging over multiple peaks' heights. Next, the signal at zero time delay (as shown in Fig. 2 of the main text) is modeled according to C. Santori et al. [9]. The values for the orthogonal bases shown in the manuscript are obtained from the model function in the parallel configuration, neglecting the interference term in the equation as in Ref. [10].

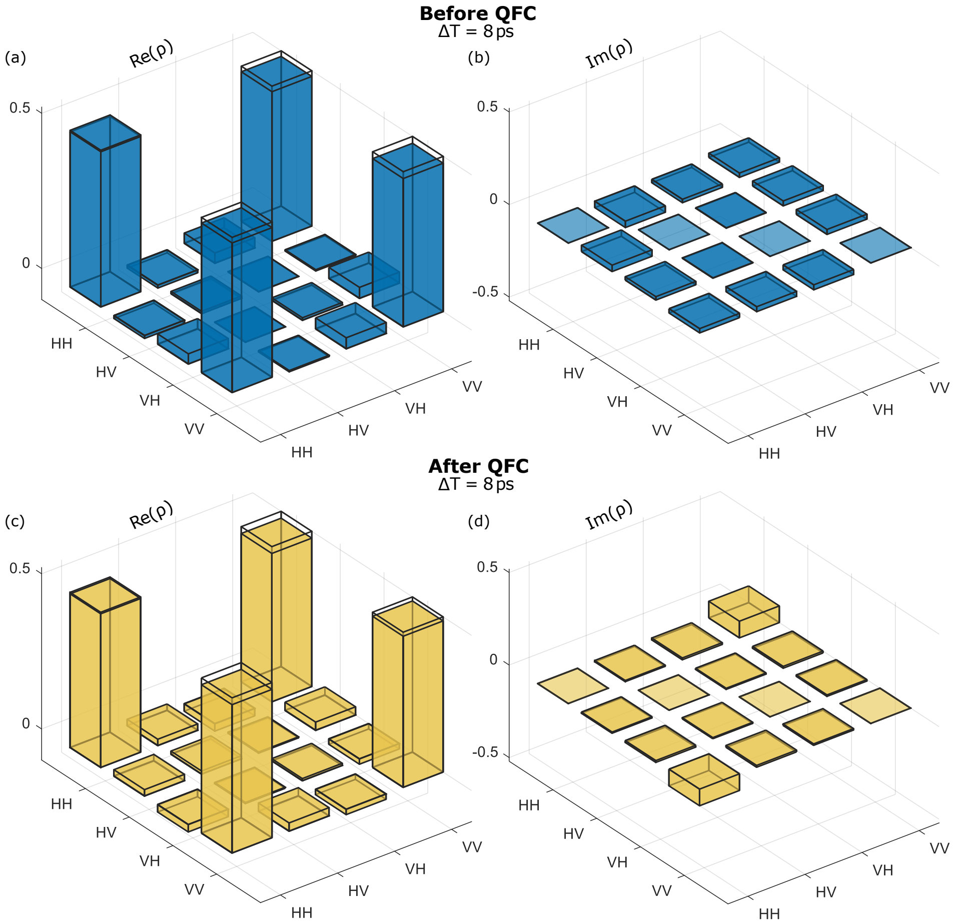

Supplementary Figure 5. **Density matrix of QD2 (a)/(b) before and (c)/(d) after conversion.**

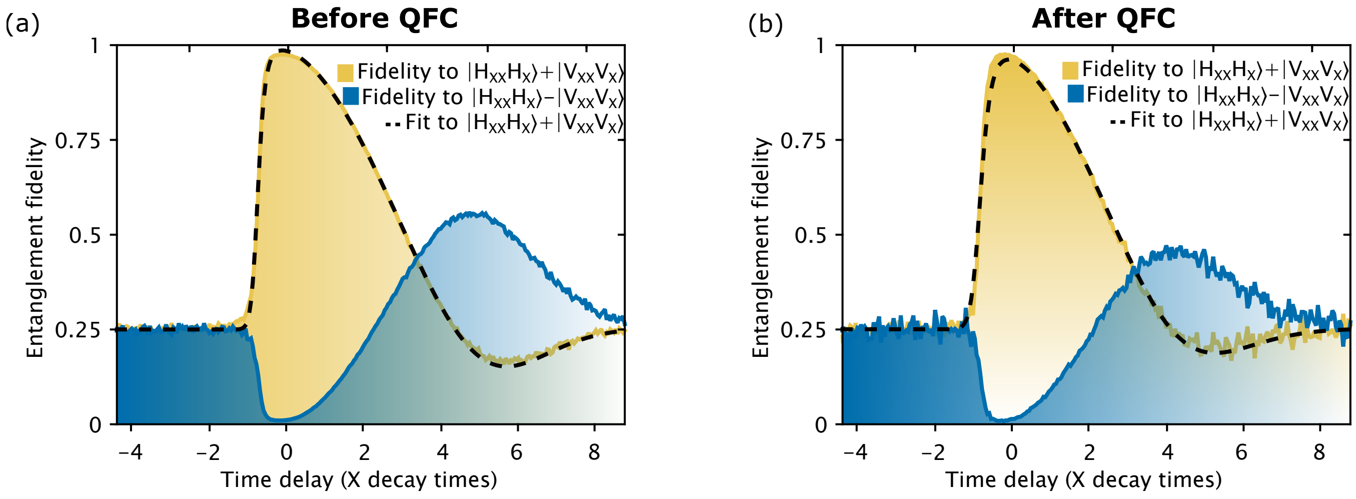

Supplementary Figure 6. **Temporal evolution of entanglement fidelity of QD2. (a)** The entanglement fidelity to $|H_{XX}H_X\rangle + |V_{XX}V_X\rangle$ (yellow) and $|H_{XX}H_X\rangle - |V_{XX}V_X\rangle$ (blue) of the two-photon state for an increasing time separation between XX and X photon is given.

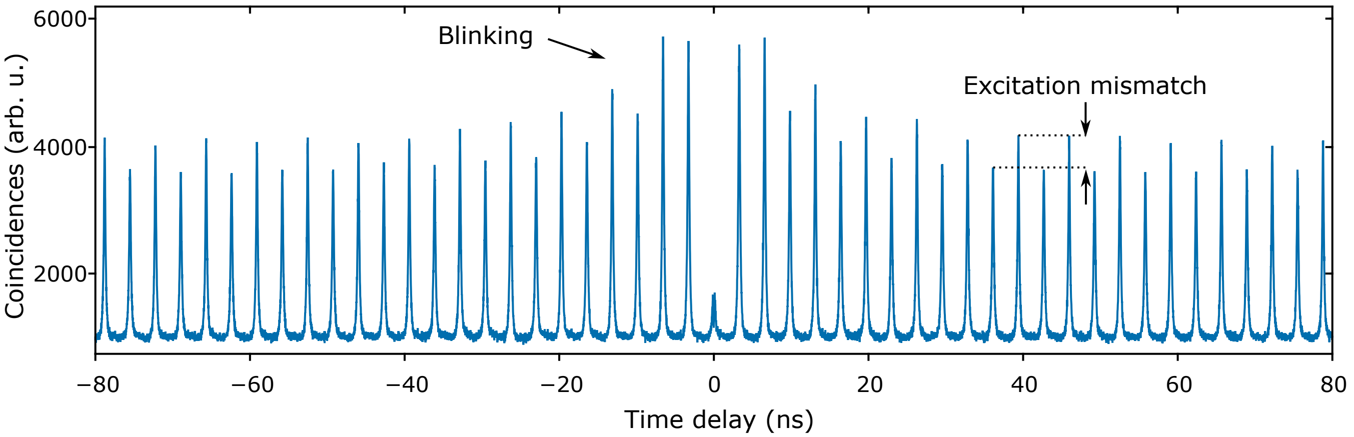

Supplementary Figure 7. **Remote two-photon-interference histogram after QFC, with parallel polarizations.**

I. Setup efficiency

An overview of all the losses connected to the various processes and optical elements is given in Supplementary Table 1. All the efficiencies directly connected to the quantum dot and the excitation are presented on the left side (laser excitation rate 304.8 MHz). On the right side, the efficiencies of the setup components and expected countrates are given. This calculation is an exemplary calculation for Photon 2 detected on detector D4. This estimation results in an overall efficiency of 0.017 %, only deviating slightly from the measured value of 0.016 % (compare Supplementary Table 2, bottom line). From these numbers, it is possible to evaluate the source brightness. As from our estimation, this also includes state preparation fidelity, excitation efficiency in presence or rate amplifier, blinking, and photonic structure extraction efficiency; we refer to it as *Total efficiency* of the source. This is estimated to be around 1.4 %. The measured countrates (averaged over all basis combinations) and deduced efficiencies are summarized in Supplementary Table 2. Notably, QD1 has a significantly lower single-photon countrate at the detector than QD2. We assign this to a lower extraction efficiency and fiber incoupling, as the QD1 emission already has a lowered countrate before conversion. Exciting both QDs with the same pulsed laser in TPE is very challenging, as the energetic TPE resonance conditions of the two dots are dissimilar, which might lead to a reduced excitation efficiency.

J. Rates and measurement times

In Supplementary Table 3 an overview of the threefold coincidences, measurement time, and threefold coincidence rates is given. The data are shown for each measurement basis combination, where the first letter is the set polarization of Photon 1, and the second letter is the polarization of the analysis setup on the receiver side of Photon 3. The given numbers belong to the data presented in the main text with Bell state $|\Psi^-\rangle_{1,2}$ and detector combination D2D4 (compare Supplementary Note 2 B) for a 290 ps time window.

Supplementary Note 2. DATA ACQUISITION AND EVALUATION

A. Temporal synchronization

For this experiment temporal synchronization in multiple parts of the experiment has to be ensured. First, photons from both QDs must arrive at the interference fiber beam splitter of the BSM simultaneously. As both QDs are excited by the same laser, the laser pulse is split into two arms, each exciting a QD respectively. An adjustable free-space path in one arm allows tuning the temporal delay between QD1 and QD2 photons for maximal overlap at the fiber beam splitter. Second, the photon detection events of X and XX photons are synchronized for all six detection channels via software delays in the time tagging unit. This makes sure that if an entangled pair of XX and X are created in the same radiative cascade they are detected at the same time. This is done to circumvent the sorting of time tags during data processing.

Supplementary Table 1. **Exemplary efficiency calculation for Photon 2 on detector D4.** (*) averaged over all four pulses. ($^{+}$) value provided by the manufacturer. (^{x}) estimation, due to a spectral uncertainty between the QD and the laser used to measure this value.

**Excitation & extraction**				**Setup**			
**Quantity**	**Efficiency**	**Cts (Hz)**		**Quantity**	**Efficiency**	**Cts (Hz)**	
State preparation	45%	1.37E+08	**Total efficiency**	Objective^{+}	85%	3.51E+06	**Total efficiency**
4-fold mismatch*	70%	9.60E+07	1.4%	Cryo window $^{+}$	90%	3.16E+06	1.2%
Blinking	43%	4.13E+07		Mirror 1	94%	2.97E+06	
Extraction [11]	10%	4.13E+06		VBG 1 (trans)	99%	2.94E+06	
				VBG 2 (trans)	99%	2.91E+06	
				LP filter	99%	2.88E+06	
				VBG 3 (reflec)	94%	2.71E+06	
				Incoupling	48%	1.30E+06	
				Fiber 1	86%	1.12E+06	
				Fiber connection	95%	1.06E+06	
				Fiber 2	95%	1.01E+06	
				QFC	47%	4.74E+05	
				Fiber BS	49%	2.32E+05	
				Spectral filtering ^{x}	65%	1.51E+05	
				Waveplates + PBS	49%	7.40E+04	
				Incoupling	90%	6.66E+04	
				Detector Pol.	90%	5.99E+04	
				Detection	85%	5.09E+04	

**Overall efficiency estimation** (Excitation, extraction and setup)
0.017%

Supplementary Table 2. **Average measured countrates and efficiencies by detection channel.** The background is already subtracted from the given photon countrates. (*) additional PBS in state preparation, ($^{+}$) no QFC.

**Detector**	**Background**	**Photon 1***		**Photon 2**		**Detector**	**Background**	**Photon 3^{+}**	
(telecom)	Avg cts (Hz)	Avg cts (Hz)	Avg eff.	Avg cts (Hz)	Avg eff.	(NIR)	Avg cts (Hz)	Avg cts (Hz)	Avg eff.
**D1**	2.54E+04	3.83E+03	0.0013%	6.40E+04	0.021%	**D5**	1.08E+05	3.84E+05	0.126%
**D2**	1.96E+04	2.33E+03	0.0008%	4.57E+04	0.015%	**D6**	7.16E+04	2.41E+05	0.079%
**D3**	1.97E+04	3.00E+03	0.0010%	4.27E+04	0.014%				
**D4**	2.13E+04	3.33E+03	0.0011%	4.79E+04	0.016%				

Supplementary Table 3. **Threefold coincidences and measurement times in for the $|\Psi^{-}\rangle_{1,2}$ state and a** 290 ps **time window.**

Basis	HH	HV	HD	HA	HR	HL	DH	DV	DD	DA	DR	DL	RH	RV	RD	RA	RR	RL
Coincidences	401	949	661	615	575	402	341	232	663	405	514	386	415	341	624	469	1024	609
Time (h)	91.8	91.8	66.0	66.0	52.5	52.5	37.0	37.0	74.0	74.0	44.9	44.9	43.9	43.9	66.9	66.9	60.2	60.2
Coinc. Rate (mHz)	1.21	2.87	2.78	2.59	3.04	2.13	2.56	1.74	2.49	1.52	3.18	2.39	2.63	2.16	2.59	1.95	4.73	2.81

B. Bell states

The BSM in the conducted experiment is performed with a polarization-selective BSM setup [12, 13]. The setup consists of a non-polarizing 50:50 fiber beam splitter and a polarizing beam splitter at each output arm. Supplementary Figure 8 depicts all possible detection outcomes depending on the projected Bell state. With the presented configuration two out of four Bell states can be identified. While the projection of states $|\Psi^{-}\rangle_{1,2}$ and $|\Psi^{+}\rangle_{1,2}$ can be resolved with the utilized detectors, states $|\Phi^{+}\rangle_{1,2}$ and $|\Phi^{-}\rangle_{1,2}$ can not be discriminated (requiring photon-number-resolving detectors). As shown in Supplementary Figure 8a there are two combinations of detector coincidences (D1D3 and D2D4) corresponding to the state $|\Psi^{-}\rangle_{1,2}$. Similarly, Supplementary Figure 8b gives two possible detector combinations (D1D2, D3D4) for the state $|\Psi^{+}\rangle_{1,2}$. In the main document of this work, the results heralded by the Bell state $|\Psi^{-}\rangle_{1,2}$ from detector coincidences D2D4 are presented. All further results are given in Supplementary Note 3. Equation 1 in the manuscript describes the three-photon state in the Bell basis.

In the following, an exemplary calculation is given for the scenario in which Photon 1 is prepared in $|H\rangle_1$. In this

case, the three-photon state becomes

$$|\Psi_{\mathrm{tot}}\rangle = |H\rangle_1 \otimes |\Phi^+\rangle_{2,3} = \frac{1}{2}(|\Phi^+\rangle_{1,2}|H\rangle_3 + |\Phi^-\rangle_{1,2}\,\sigma_3\,|H\rangle_3 + |\Psi^+\rangle_{1,2}\,\sigma_1\,|H\rangle_3 - |\Psi^-\rangle_{1,2}\,\sigma_1\sigma_3\,|H\rangle_3). \tag{9}$$

Assuming Photon 1 and Photon 2 herald the Bell state $|\Psi^-\rangle_{1,2}$, Photon 3 will be projected onto state $|V\rangle_3$:

$$-\,|\Psi^-\rangle_{1,2}\,\sigma_1\sigma_3\,|H\rangle_3 = |\Psi^-\rangle_{1,2}\,|V\rangle_3\,. \tag{10}$$

The initial state is obtained by applying a unitary transformation to Photon 3, corresponding to the inverse of the two Pauli matrices $\sigma_1\sigma_3$:

$$-\sigma_3^{-1}\sigma_1^{-1}\,|V\rangle_3 = \sigma_3\sigma_1\,|V\rangle_3 = |H\rangle_3\,. \tag{11}$$

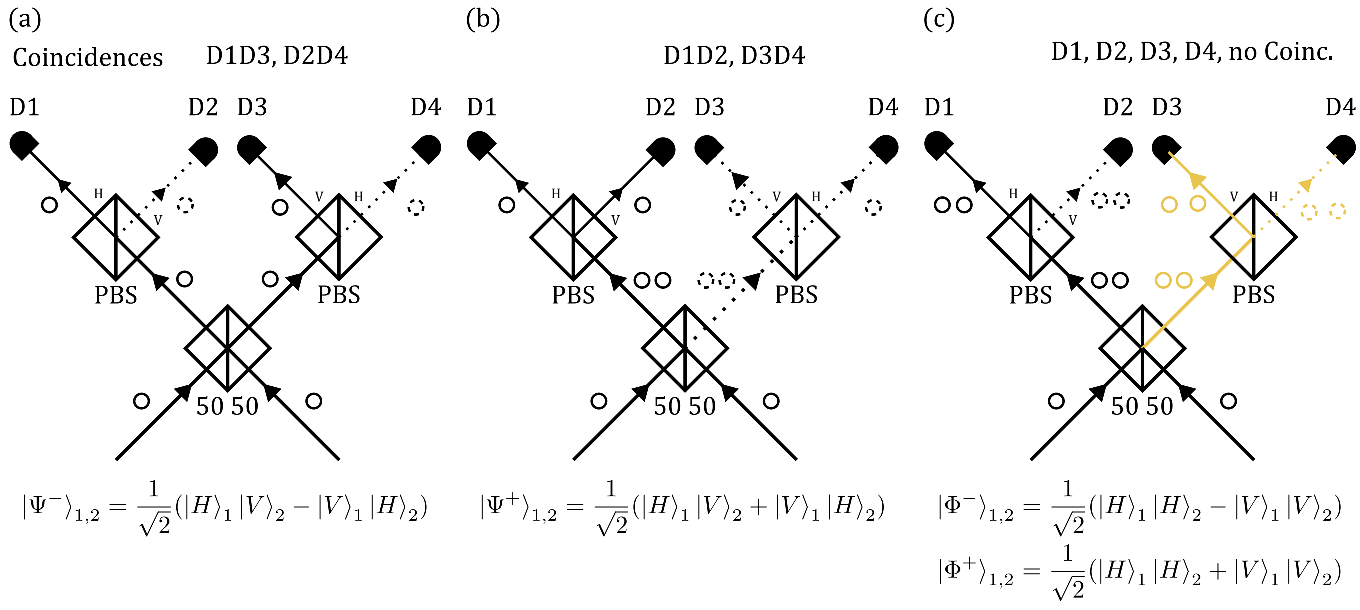

Supplementary Figure 8. **Possible Bell state projections in the polarization-selective BSM setup.** A 50:50 beam splitter followed by two polarizing beam splitters allows the identification of different Bell states. On the top, the different detector combinations corresponding to a certain Bell state projection are given. Below, the propagation of photons (solid or dashed dots) through the setup is shown followed by the corresponding Bell state. The two states given in **(a)** and **(b)** can be detected and discriminated. The $|\Phi^{\pm}\rangle_{1,2}$ states shown in **(c)** can not be identified in this experiment, requiring photon-number-resolving detectors.

C. Threefold coincidences

In this subsection, the collection, processing, and evaluation of recorded time tags is discussed.
During the teleportation experiment time tags on all four telecom detection channels in the BSM and two NIR channels on the receiver side are recorded with the Time Tagger Ultra model of Swabian Instruments . The synchronization discussed in Supplementary Note 2 A ensures that all photons detected simultaneously belong to the same experimental repetition. After the data collection is complete threefold coincidences between i) a two-fold coincidence of the BSM (as in Supplementary Figure 8a and b) and ii) a channel on the 780 nm receiver side are found. For this, all possible combinations of threefold coincidences from clicks of the three channels within a certain window are determined. Next, each coincidence is assigned to a bin of a 2D histogram based on the respective time difference between the detection

of Photon 1 and Photon 2 compared to Photon 3. An example of the resulting 2D histogram for the teleportation of state $|R\rangle$ projected onto $|R\rangle$ ($|L\rangle$) on the receiver side, heralded by $|\Psi^-\rangle_{1,2}$, and detector combination D2D4 is given in Supplementary Figure 9a,c (b,d). The figure shows the number of threefold coincidences (color code) versus the time difference between the receiver photon (Photon 3) and the first photon of the BSM (Photon 1) on the x-axis, and the time difference between the receiver photon (Photon 3) and the second photon of the BSM (Photon 2) on the y-axis. For each input polarization state $|\xi\rangle_1$, each BSM detector combination and receiver polarization projection a 2D map is created. The visible grid of equidistant bright peaks in Supplementary Figure 9a and b has a period of 3.28 ps given by the laser excitation rate. The most unique and relevant point is the central peak (highlighted by a yellow box in Supplementary Figure 9c and d) at zero time delay. At this point, all three photons belonging to the same experimental repetition coincide. The dimmer diagonal peaks going from the bottom left to the top right correspond to the situation where Photon 1 and Photon 2 are detected simultaneously and Photon 3 multiple repetitions later. On the horizontal (vertical) Photon 3 and Photon 2 (Photon 1) arrive simultaneously and Photon 1 (Photon 2) multiple repetitions later. At all remaining points all three photons are delayed to each other. All off-diagonal points are brighter than the diagonal points because they can also occur from EPS photon coincidences only. At all these points all three detected photons may stem from the EPS created in an earlier or later repetition causing false coincidences. This scenario is very likely as the EPS is brighter than the SPS. On the diagonal, the third photon cannot come from the EPS. For this reason, the diagonal side peaks are used to normalize the central peak. The normalization is done with 32 peaks at time differences beyond any blinking timescale. After normalization, the number of coincidences inside the center window is extracted for further evaluation. A low amount of continuous wave (CW) above-barrier excitation (for stabilizing the charge environment of the QDs) leads to a residual stream of CW-entangled photons from the EPS, resulting in weak continuous horizontal and vertical lines.

D. Monte-Carlo simulation

The Monte-Carlo simulation employed to retrieve the error bars given in Fig. 3 and Fig. 4 of the main text is explained in the following. First, the number of threefold coincidences N_{A-B} for a given basis combination $A-B$ and time window Δt is determined. Second, assuming Poissonian counting statistics, the threefold coincidences are modeled by a Poissonian probability distribution function $P(N_{A-B})$ with center N_{A-B}. This results in a probability distribution function for each basis combination. Third, threefold coincidences are randomly picked from each distribution and then evaluated with a maximum-likelihood method to estimate the corresponding density matrix. Fourth, the fidelities of the obtained density matrix are calculated. Fifth, the third and fourth step is repeated 10 000 times, receiving a distribution of density matrices and fidelities, as shown in Supplementary Figure 10. One standard deviation of the shown distributions is used as an error bar for data shown in Fig. 3a-c of the main text. This process is repeated for all input states and time windows. Gaussian propagation of standard deviations (calculated from the three teleportation measurements) is performed to obtain the average teleportation fidelity error bars.

Supplementary Note 3. TELEPORTATION WITH FURTHER BELL STATES

The density matrices of the measured teleported state (before any unitary operation), heralded by all Bell states detected across all detector combinations as described in Supplementary Note 2 C, are shown in Supplementary Figure 11a-f and Supplementary Figure 12a-f. These figures also depict the corresponding theoretically calculated density matrices in g-i respectively. All density matrices belonging to the same teleported state show similar quantitative behavior (a discussion of this behavior is found in the main text) and agree well with the theoretical model (see also Supplementary Note 4 B). Comparing the imaginary off-diagonals Im $\left(\rho_{HV/VH}\right)$ in c, f, and i between the $|\Psi^-\rangle_{1,2}$ and $|\Psi^+\rangle_{1,2}$ states the signs are flipped. This is because the matrices are shown before any unitary transformation leading to a different sign depending on the measured Bell state. Fidelities of the teleported state $|\xi\rangle_3$ (heralded by all Bell states detected across all detector combinations) to the three conjugate input states are shown in Supplementary Figure 13 and Supplementary Figure 14. Similar to the results in the main text the teleportation of the $|H\rangle$ polarization state has the highest fidelity with values between 75 % and 90 % for a 70 ps time window (depending on the detector combination) slightly dropping for larger time windows. For most of the given data, the fidelity to the expected polarization state is the highest (i.e. $|H\rangle$ in Supplementary Figure 13a, $|D\rangle$ in b and $|R\rangle$ in c).
The individual output state fidelities were used to calculate the average teleportation fidelity, which is defined as $\bar{f} = \frac{1}{3}\left(f^{|H\rangle\rightarrow|H\rangle} + f^{|D\rangle\rightarrow|D\rangle} + f^{|R\rangle\rightarrow|R\rangle}\right)$, see Supplementary Figure 15a-d. The total average teleportation fidelity was determined by first calculating the average teleportation fidelity for each Bell state, considering all possible detector combinations. These individual averages were then combined using the arithmetic mean to obtain the total

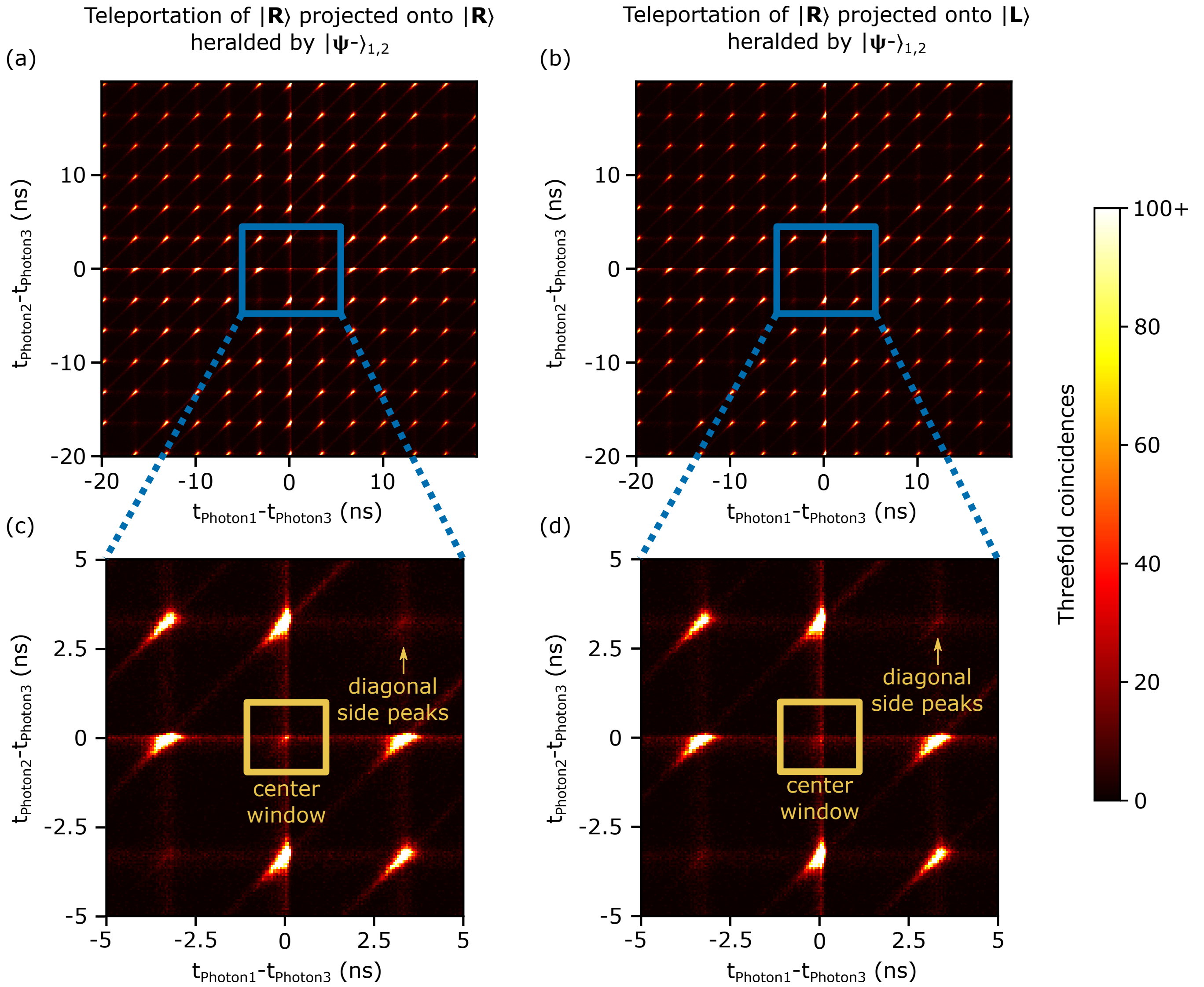

Supplementary Figure 9. **Threefold coincidence histogram.** Threefold coincidences between two BSM detector channels heralding a Bell state and Photon 3 projected onto state $|R\rangle$ and $|L\rangle$ in **(a)** and **(b)** on the receiver side. In **(c)** and **(d)** a zoom-in of the center region at zero delay including the central coincidence peak used for the density matrix reconstruction is depicted.

average fidelity which is shown in Supplementary Figure 15e. The fidelities heralded by the $|\Psi^{\pm}\rangle_{1,2}$ states given in c and d (detector combination D3D4 and D2D4) show higher values than the fidelities in a and b (combinations D1D2 and D1D3). For short time windows c and d are significantly above the classical threshold, while for a and b the classical threshold is within the error bar. We attribute this behavior to setup-related imperfections in the detection path of D1. However, the arithmetic mean over all detector combinations in e shows teleportation fidelities significantly above the classical threshold for up to 100 ps time windows, validating the overall success of the experiment.

Supplementary Note 4. THEORETICAL DESCRIPTION

A. Modeling

The theoretical model to describe the output state of the teleportation protocol is based on the process matrix formalism, which was for teleportation with quantum dot single-photon sources described in [12, 14].

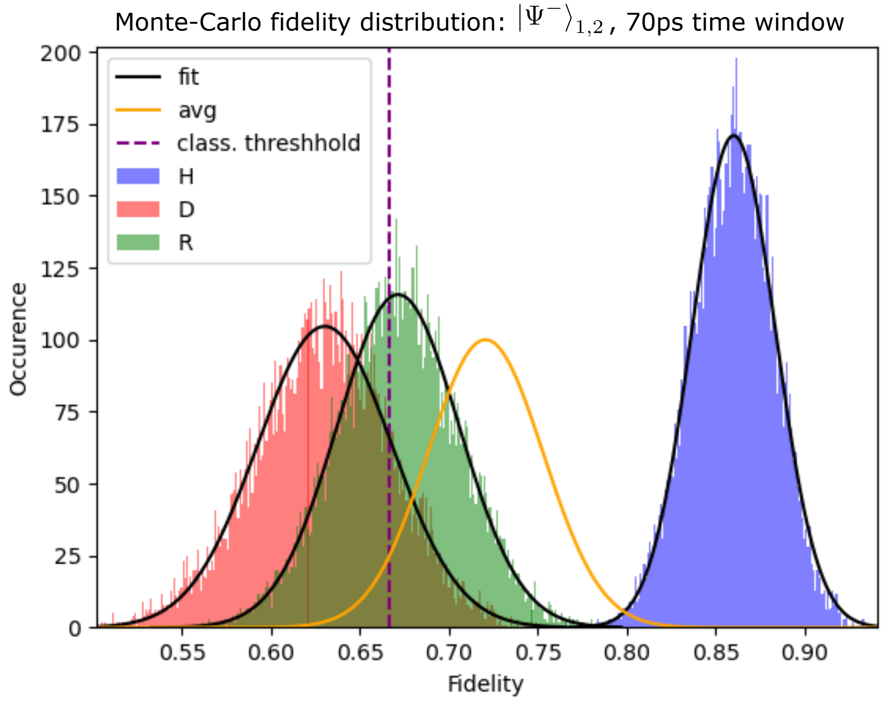

Supplementary Figure 10. **Distribution of fidelities from Monte-Carlo simulation.** The blue (red, green) distribution shows the fidelity distribution when teleporting $|H\rangle$ ($|D\rangle$, $|R\rangle$). The data are obtained via a Monte-Carlo simulation with 10 000 runs based on a 70 ps histogram window when projecting onto Bell state $|\Psi^-\rangle_{1,2}$ with detector combination D2D4. Solid black lines are a Gaussian fit to the data. The average teleportation fidelity is given as a yellow solid line. The center of the yellow curve is the arithmetic mean of the three black distributions and the width is calculated via Gaussian propagation of standard deviations (also calculated from the three teleportation measurements).

$$\hat{\rho}^{\psi^\pm}_{\text{teleported}}(|\psi_{\text{in}}\rangle) = \frac{1}{2}\begin{bmatrix} 1 - c^{'}kg^{'(1)}_{\text{HV}}\left(|\,\alpha\,|^2 - |\,\beta\,|^2\right) & \pm\frac{2\alpha^*\beta kcg^{(1)}_{\text{HV}}}{\sqrt{1+\left(\frac{S\tau_X}{\hbar}g^{(1)}_{\text{HV}}\right)^2\left(1+\left(\frac{S\tau_X}{\hbar}g^{(1)}_{\text{deph}}\right)^2\right)}} \\ \pm\frac{2\alpha\beta^* kcg^{(1)}_{\text{HV}}}{\sqrt{1+\left(\frac{S\tau_X}{\hbar}g^{(1)}_{\text{HV}}\right)^2\left(1+\left(\frac{S\tau_X}{\hbar}g^{(1)}_{\text{deph}}\right)^2\right)}} & 1 + c^{'}kg^{'(1)}_{\text{HV}}\left(|\,\alpha\,|^2 - |\,\beta\,|^2\right) \end{bmatrix}, \tag{12}$$

where complex amplitudes α and β define an arbitrary input state described as $|\psi_{\text{in}}\rangle = \alpha\,|H\rangle + \beta\,|V\rangle$, S is the amplitude of FSS (of QD2), τ_X is a lifetime of excitonic state, k is the k-factor as described in Supplementary Note 1 F, c and $c^{'}$ are coefficients depending on the implementation of the Bell state measurement, $g^{(1)}_{\text{HV}}$ is the first-order cross-coherence, $g^{'(1)}_{\text{HV}}$ is a fraction of dot emission unaffected by spin-scattering, which is equivalent to $g^{(1)}_{\text{HV}}$ in the absence of cross-dephasing and $g^{(1)}_{\text{deph}}$ first-order coherence. The first-order coherences are defined as follows

$$\begin{aligned} g^{(1)}_{\text{HV}} &= \frac{1}{1+\tau_X/\tau_{\text{SS}}+\tau_X/\tau_{\text{HV}}}\,, \\ g^{'(1)}_{\text{HV}} &= \frac{1}{1+\tau_X/\tau_{\text{HV}}}\,, \\ g^{(1)}_{\text{deph}} &= \frac{1}{1+2\tau_X/T_2}\,, \end{aligned} \tag{13}$$

where τ_{SS} is spin scattering time, τ_{HV} is cross dephasing time and T_2 is dephasing time. For implementations of a Bell state measurement, where polarization-sensitive elements are used (as in this work), the coefficient c and $c^{'}$ are defined as [12]

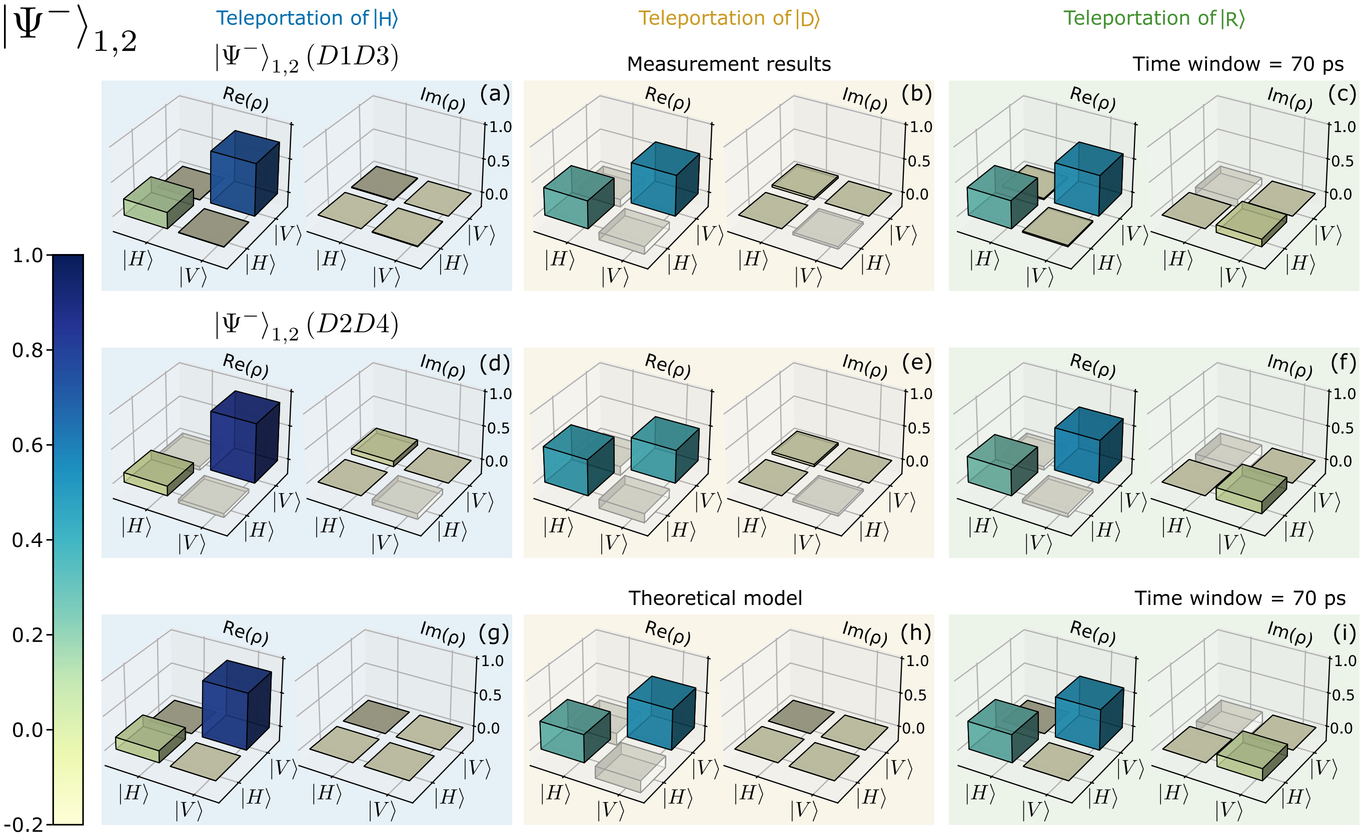

Supplementary Figure 11. **Density matrices of the measured teleported state heralded by Bell state $|\Psi^-\rangle_{1,2}$ in comparison to corresponding theoretically calculated density matrices.** Real and imaginary parts of the density matrix of the teleported polarization state $-\sigma_1\sigma_3\,|\xi\rangle_3$ at a 70 ps time window obtained from the measurement data heralded by Bell state $|\Psi^-\rangle_{1,2}$ detected by detectors $D1D3/D2D4$ before any unitary operation are shown for the following input states: **(a)/(d)** $|H\rangle$, **(b)/(e)** $|D\rangle$, **(c)/(f)** $|R\rangle$. The theoretically obtained density matrices of teleported states are shown for the same input states in **(g)**, **(h)**, and **(i)**. The following parameters were assumed in the theory model: polarization mode overlap $M_p = 0.85$, dephasing time $T_2 = 35\,\text{ps}$, cross-dephasing time τ_{HV}=5 ns, spin scattering time $\tau_{ss} = 5\,\text{ns}$, lifetime of exciton $\tau_X = 171\,\text{ps}$, TPI visibility $V = 79\,\%$ and ratio of true three-fold coincidences $k = 0.85$.

$$\begin{aligned} c &= V\ , \\ c' &= 1\ , \end{aligned} \tag{14}$$

where V is the TPI visibility. Based on ref. [14] the values of cross-dephasing time and spin scattering time were assumed within uncertainty intervals $\tau_{HV} = [1, 10]\,\text{ns}$ and $\tau_{ss} = [1, 10]\,\text{ns}$ respectively. These values should well describe GaAs QDs obtained by droplet epitaxy[14]. The dephasing time was calculated from the measured linewidth of the exciton spectral line as $T_2 = 35\,\text{ps}$. The dephasing time ($T_2 = 35\,\text{ps}$) was derived from the measured linewidth of the exciton spectral line, $\Delta\lambda_X = 5.0\,\text{GHz}$. This linewidth consists of a natural Lorentzian component with linewidth 0.93 GHz, corresponding to the Fourier-limited spectral linewidth of the measured exciton lifetime ($\tau_X = 171\,\text{ps}$), and a Gaussian component representing the inhomogeneous broadening due to dephasing processes. Through spectral deconvolution, we determined that the inhomogeneous broadening component corresponds to a linewidth of 4.50 GHz, which translates to a dephasing time of approximately 35 ps. This value was therefore used as the dephasing time T_2 in our model. The k-factor and TPI visibility were obtained from characterization measurements, see Supplementary Note 1 F and Supplementary Note 1 H.

In the theoretical framework outlined by [12], it is assumed that the spectral splitting of Photon 2 between the $|H\rangle$ and $|V\rangle$ wave packets, given by nonzero FSS, reduces the TPI visibility. This results in a mixing of the output state and lowers the teleportation fidelity. Here we assume a specific alignment procedure of the experimental setup, as employed, where the TPI visibility was maximized by optimizing spectral and temporal overlap between the Photon 1

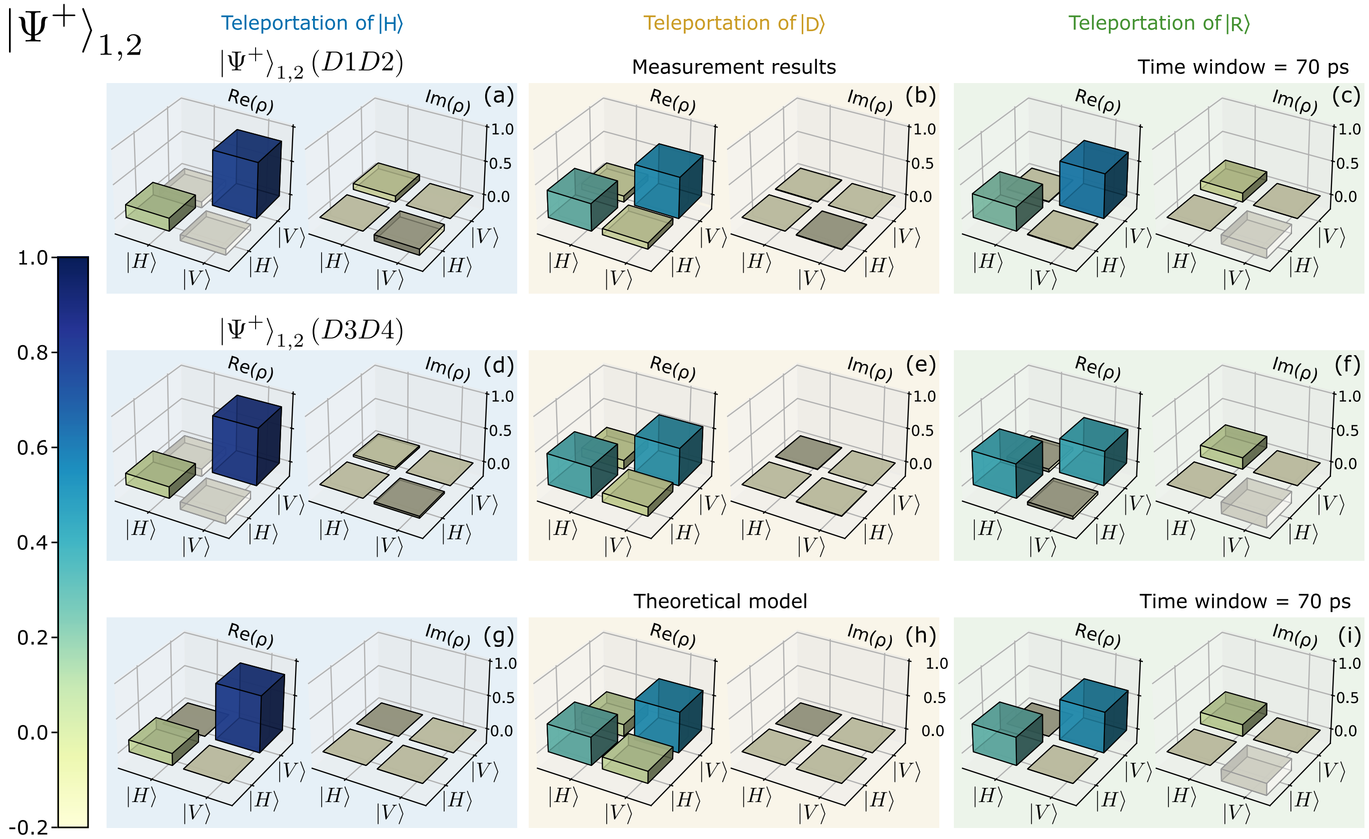

Supplementary Figure 12. **Density matrices of the measured teleported state heralded by Bell state $|\Psi^+\rangle_{1,2}$ in comparison to corresponding theoretically calculated density matrices.** Real and imaginary parts of the density matrix of the teleported state $\sigma_1\,|\xi\rangle_3$ at a 70 ps time window obtained from the measurement data heralded by Bell state $|\Psi^+\rangle_{1,2}$ detected by detectors $D1D2/D3D4$ before any unitary operation are shown for the following input states: **(a)/(d)** $|H\rangle$, **(b)/(e)** $|D\rangle$, **(c)/(f)** $|R\rangle$. The theoretically obtained density matrices of teleported states are shown for the same input states in **(g)**, **(h)**, and **(i)**. The following parameters were assumed in the theory model: polarization mode overlap $M_p = 0.85$, dephasing time $T_2 = 35\,\text{ps}$, cross-dephasing time τ_{HV}=5 ns, spin scattering time $\tau_{ss} = 5\,\text{ns}$, lifetime of exciton $\tau_X = 171\,\text{ps}$, TPI visibility $V = 79\,\%$ and ratio of true three-fold coincidences $k = 0.85$.

and Photon 2 for fixed $|H\rangle$ polarization. Therefore the measured V describes the TPI visibility between two $|H\rangle$ wave packets and the TPI visibility for $|H\rangle$ and $|V\rangle$ wave packets overlap is then given as

$$V_{\text{overlap}} = M_p V \ , \tag{15}$$

where M_p is the polarization mode overlap, describing the spectral overlap between $|H\rangle$ and $|V\rangle$ wave packets. Therefore the TPI visibility is in the end higher for the $|H\rangle$ polarized wave packet than for the $|V\rangle$ polarized one. From now we will assume $c = V_{\text{overlap}}$ and $c^{'} = 1$ as only interference between the overlapped wavepackets is constructively contributing to the teleportation process.

With a probability $(1 - M_p)$ the general input state $|\psi_{\text{in}}\rangle = \alpha\,|H\rangle + \beta\,|V\rangle$ will selectively interfere with the $|H\rangle$ wavepacket only. In this case, we can assume that the output state described by 12 for $|\psi_{\text{in}}\rangle = 1\,|H\rangle + 0\,|V\rangle$ as

$$\hat{\rho}^{\psi^\pm}_{\text{out}}(|H\rangle) = \frac{1}{2}\begin{bmatrix} 1 - c^{'}kg^{'(1)}_{\text{HV}} & 0 \\ 0 & 1 + c^{'}kg^{'(1)}_{\text{HV}} \end{bmatrix} \ , \tag{16}$$

where $c^{'} = 1$. The full output state of the teleportation protocol is then given as the following state mixture

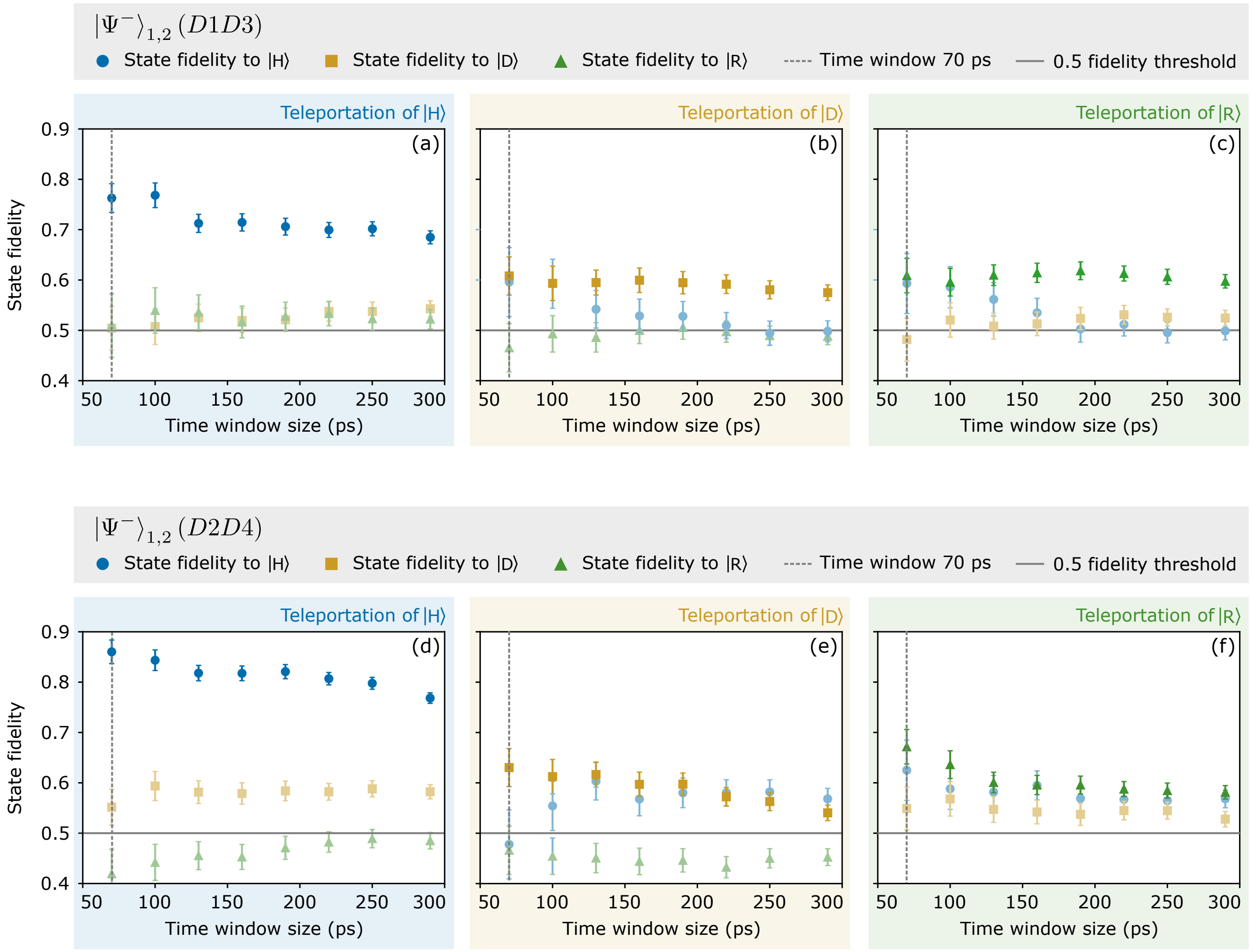

Supplementary Figure 13. **Teleportation of three conjugate polarization states heralded by Bell state $|\Psi^-\rangle_{1,2}$:** The teleportation experiment is repeated with three conjugate input states of Photon 1: $|\xi\rangle_1 = |H\rangle$, $|D\rangle$ and $|R\rangle$. The fidelities of the output state $|\xi\rangle_3$ heralded by Bell state $|\Psi^-\rangle_{1,2}$ detected by detectors $D1D3/D2D4$ are shown for the following input states $|\xi\rangle_1$: **(a)/(d)** $|H\rangle$, **(b)/(e)** $|D\rangle$, **(c)/(f)** $|R\rangle$. The fidelity to the three ideal polarization states $|H\rangle$, $|D\rangle$ and $|R\rangle$ is shown in blue, yellow, and green. The error bars correspond to one standard deviation. The solid grey line at 1/2 symbolizes zero degree of polarization in the respective basis.

$$\hat{\rho}_{\text{out}}(|\psi_{\text{in}}\rangle) = \frac{1}{2}\begin{bmatrix} M_p\left(1 - k g'^{(1)}_{\text{HV}}\left(|\,\alpha\,|^2 - |\,\beta\,|^2\right)\right) & \pm\frac{2\alpha^*\beta k M_p V g^{(1)}_{\text{HV}}}{\sqrt{1+\left(\frac{S\tau_X}{\hbar}g^{(1)}_{\text{HV}}\right)^2}\left(1+\left(\frac{S\tau_X}{\hbar}g^{(1)}_{\text{deph}}\right)^2\right)} \\ \pm\frac{2\alpha\beta^* k M_p V g^{(1)}_{\text{HV}}}{\sqrt{1+\left(\frac{S\tau_X}{\hbar}g^{(1)}_{\text{HV}}\right)^2}\left(1+\left(\frac{S\tau_X}{\hbar}g^{(1)}_{\text{deph}}\right)^2\right)} & M_p\left(1 + k g'^{(1)}_{\text{HV}}\left(|\,\alpha\,|^2 - |\,\beta\,|^2\right)\right) \end{bmatrix} + \frac{1}{2}(1 - M_p)\begin{bmatrix} 1 - k g'^{(1)}_{\text{HV}} & 0 \\ 0 & 1 + k g'^{(1)}_{\text{HV}} \end{bmatrix}. \tag{17}$$

Since, for QDs, the coefficients k and $g'^{(1)}_{\text{HV}}$ are close to 1 [12] and for low FSS the polarization mode overlap

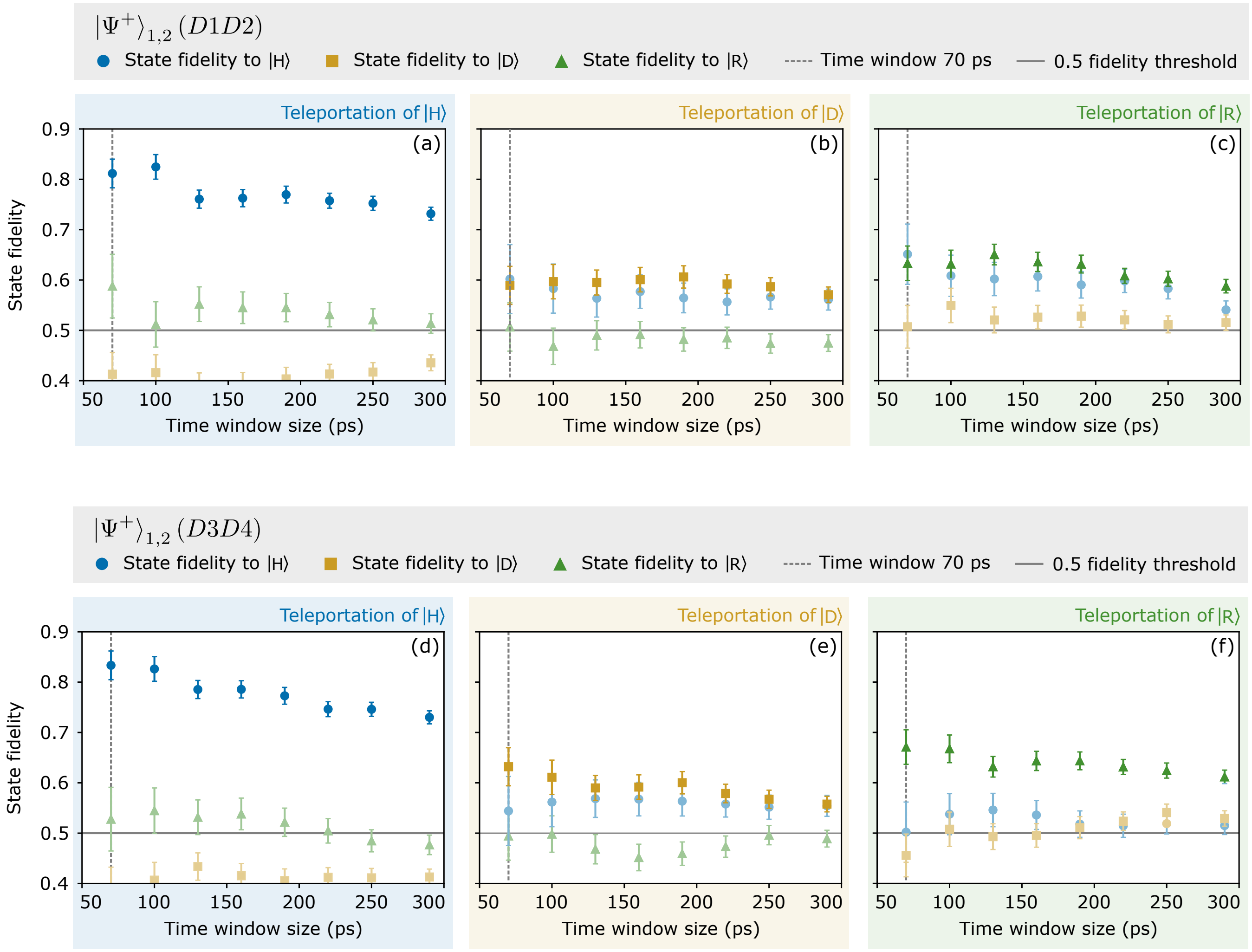

Supplementary Figure 14. **Teleportation of three conjugate polarization states heralded by Bell state $|\Psi^+\rangle_{1,2}$:** The teleportation experiment is repeated with three conjugate input states of Photon 1: $|\xi\rangle_1 = |H\rangle$, $|D\rangle$ and $|R\rangle$. The fidelities of the output state $|\xi\rangle_3$ heralded by Bell state $|\Psi^+\rangle_{1,2}$ detected by detectors $D1D2/D3D4$ are shown for the following input states $|\xi\rangle_1$: **(a)/(d)** $|H\rangle$, **(b)/(e)** $|D\rangle$, **(c)/(f)** $|R\rangle$. The fidelity to the three ideal polarization states $|H\rangle$, $|D\rangle$ and $|R\rangle$ is shown in blue, yellow and green. The error bars correspond to one standard deviation. The solid grey line at 1/2 symbolizes zero degree of polarization in the respective basis.

approaches $M_p \to 1$, we can assume in the second term that $(1 - M_p) \to 0$ and therefore approximate $k \approx 1$ and $g_{\mathrm{HV}}^{'(1)} \approx 1$. Now we can write the output state in a simpler form

$$\hat{\rho}_{\mathrm{out}}^{\psi^\pm}(|\psi_{\mathrm{in}}\rangle) = M_p \hat{\rho}_{\mathrm{teleported}}^{\psi^\pm}(|\psi_{\mathrm{in}}\rangle) + (1 - M_p)|V\rangle\langle V| \ . \tag{18}$$

It is important to note that the additional $|V\rangle\langle V|$ term in the teleportation output state is specific to the chosen alignment procedure, representing a worst-case scenario. Here, the TPI visibility is maximized only for one of the polarized wave packets—in this case, the $|H\rangle$ polarized packet. The significance of this term would be reduced if the TPI visibility were instead maximized for either the $|D\rangle = \frac{1}{\sqrt{2}}|H\rangle + \frac{1}{\sqrt{2}}|V\rangle$ or $|A\rangle = \frac{1}{\sqrt{2}}|H\rangle - \frac{1}{\sqrt{2}}|V\rangle$ polarization states. In such scenario, the spectral splitting of the $|H\rangle$ and $|V\rangle$ polarized wave packets would not favor one wave packet over the other in the teleportation protocol; it would just mix the output state and therefore reduce the teleportation fidelity as already described by $\hat{\rho}_{\mathrm{teleported}}(|\psi_{\mathrm{in}}\rangle)$.

The polarization mode overlap can be calculated from the FSS and spectral linewidth of Photon 2. The M_p for given experimental parameters, FSS amplitude 2.1 µeV and linewidth 4.3 GHz, was calculated by numerical integration as

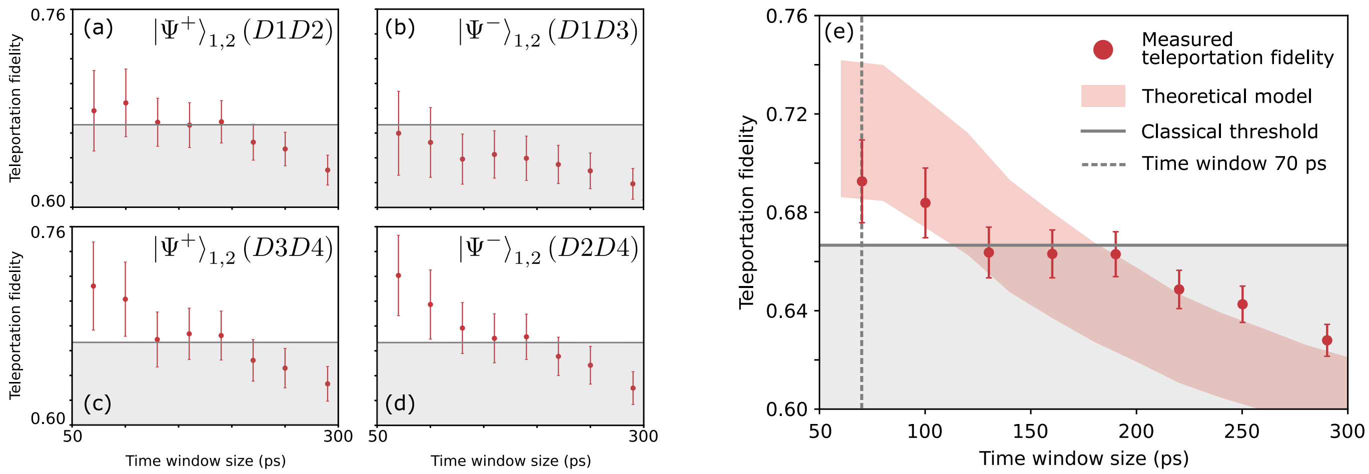

Supplementary Figure 15. **Average teleportation fidelity**: The average fidelity of all three performed teleportation experiments $\bar{f} = (f^{|H\rangle \to |H\rangle} + f^{|D\rangle \to |D\rangle} + f^{|R\rangle \to |R\rangle})/3$, heralded by: **(a)** Bell state $|\Psi^+\rangle_{1,2}$ detected by detectors $D1D2$, **(b)** Bell state $|\Psi^+\rangle_{1,2}$ detected by detectors $D3D4$, **(c)** Bell state $|\Psi^-\rangle_{1,2}$ detected by detectors $D1D3$, **(d)** Bell state $|\Psi^-\rangle_{1,2}$ detected by detectors $D2D4$. Red solid dots represent the measured results with error bars corresponding to one standard deviation. The grey solid line symbolizes the classical threshold of 2/3. **(e)** The total average teleportation fidelity was calculated as the arithmetical mean over the individual averages in **(a)**-**(d)** (all possible detector combinations). The red-shaded area shows the theoretically modelled data. The uncertainty in the theoretical model arises from some parameters that cannot be precisely determined. These parameters are instead estimated within specific intervals to account for their variability: polarization mode overlap $M_p = [0.8, 0.9]$, cross-dephasing time $\tau_{HV} = [1, 10]$ ns, spin scattering time $\tau_{ss} = [1, 10]$ ns. The other parameters are assumed as: dephasing time $T_2 = 35$ ps and lifetime of exciton $\tau_X = 171$ ps.

$M_p = 0.94$. Because of the uncertainty in the impact of setup birefringence on M_p, the value of the polarization mode overlap was assumed within the interval $M_p = [0.8, 0.9]$ in most of the calculations.

B. Fidelities to measurements

To demonstrate the agreement between experimentally measured teleported states and the output states theoretically predicted by the model, the corresponding fidelities were calculated as

$$F\left(\hat{\xi}_3(|\psi_{\rm in}\rangle), \hat{\rho}_{\rm out}(|\psi_{\rm in}\rangle)\right) = \mathrm{Tr}\left[\sqrt{\sqrt{\hat{\xi}_3(|\psi_{\rm in}\rangle)}\hat{\rho}_{\rm out}(|\psi_{\rm in}\rangle)\sqrt{\hat{\xi}_3(|\psi_{\rm in}\rangle)}}\right]^2 , \quad (19)$$

where $\hat{\xi}_3(|\psi_{\rm in}\rangle)$ is experimentally measured output state density matrix and $\hat{\rho}_{\rm out}(|\psi_{\rm in}\rangle)$ is density matrix obtained by the theoretical model given by Supplementary Equation 18.

In Supplementary Figure 16, the fidelity of the measured teleported state heralded by Bell state $|\Psi^-\rangle_{1,2}$ (detector combination D2D4) to the corresponding theoretically predicted output states is shown. The corresponding density matrices for a 70 ps time window are presented in Fig. 3 of the main text. The following parameters were assumed in the theory model: polarization mode overlap $M_p = 0.85$, dephasing time $T_2 = 35$ ps, cross-dephasing time τ_{HV}=5 ns, spin scattering time $\tau_{ss} = 5$ ns, lifetime of exciton $\tau_X = 171$ ps, TPI visibility $V = 79\,\%$ and ratio of true three-fold coincidences $k = 0.85$.

The calculated fidelities exceed 0.96, indicating good agreement between the theoretical model and experimental results. For the input states $|D\rangle$ and $|R\rangle$, fidelities are approximately 0.99. The fidelity for $|H\rangle$ is reduced due to the polarization mode overlap uncertainty, estimated as the mean value within the interval $M_p = [0.8, 0.9]$. Reduced fidelities observed for shorter time windows ($\approx$ 60 ps) are attributed to the limited time resolution of the employed detectors and lower signal-to-noise ratio at these shorter intervals.

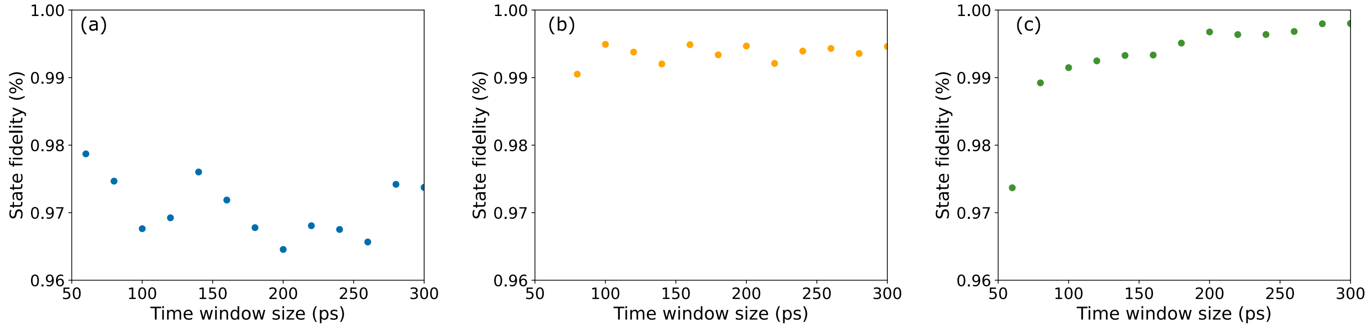

Supplementary Figure 16. **Fidelity of experimentally measured output state** $\hat{\xi}_3(|\psi_{\rm in}\rangle)$ (heralded by Bell state $|\Psi^-\rangle_{1,2}$ with detector combination D2D4) in reference to the output obtained by the theory model $\hat{\rho}_{\rm out}(|\psi_{\rm in}\rangle)$ for the following input states $|\psi_{\rm in}\rangle$: **(a)**
$|H\rangle$, **(b)** $|D\rangle$, **(c)** $|R\rangle$. The following parameters were assumed in the theory model: polarization mode overlap $M_p = 0.85$, dephasing time $T_2 = 35\,$ps, cross-dephasing time τ_{HV}=5 ns, spin scattering time $\tau_{ss} = 5\,$ns, lifetime of exciton $\tau_X = 171\,$ps, TPI visibility $V = 79\,\%$ and ratio of true three-fold coincidences $k = 0.85$.

[1] A. V. Kuhlmann, J. Houel, A. Ludwig, L. Greuter, D. Reuter, A. D. Wieck, M. Poggio, and R. J. Warburton, Charge noise and spin noise in a semiconductor quantum device, Nature Physics **9**, 570 (2013).
[2] H. Vural, S. L. Portalupi, and P. Michler, Perspective of self-assembled ingaas quantum-dots for multi-source quantum implementations, Applied Physics Letters **117** (2020).
[3] T. Strobel, J. H. Weber, M. Schmidt, L. Wagner, L. Engel, M. Jetter, A. D. Wieck, S. L. Portalupi, A. Ludwig, and P. Michler, A unipolar quantum dot diode structure for advanced quantum light sources, Nano Letters **23**, 6574 (2023).
[4] M. Vyvlecka, L. Jehle, C. Nawrath, F. Giorgino, M. Bozzio, R. Sittig, M. Jetter, S. L. Portalupi, P. Michler, and P. Walther, Robust excitation of C-band quantum dots for quantum communication, Applied Physics Letters **123**, 174001 (2023).
[5] A. J. Hudson, R. M. Stevenson, A. J. Bennett, R. J. Young, C. A. Nicoll, P. Atkinson, K. Cooper, D. A. Ritchie, and A. J. Shields, Coherence of an entangled exciton-photon state, Phys. Rev. Lett. **99**, 266802 (2007).
[6] R. Winik, D. Cogan, Y. Don, I. Schwartz, L. Gantz, E. R. Schmidgall, N. Livneh, R. Rapaport, E. Buks, and D. Gershoni, On-demand source of maximally entangled photon pairs using the biexciton-exciton radiative cascade, Phys. Rev. B **95**, 235435 (2017).
[7] T. Müller, J. Skiba-Szymanska, A. B. Krysa, J. Huwer, M. Felle, M. Anderson, R. M. Stevenson, J. Heffernan, D. A. Ritchie, and A. J. Shields, A quantum light-emitting diode for the standard telecom window around 1,550nm, Nat. Commun. **9**, 862 (2018).
[8] T. Strobel, S. Kazmaier, T. Bauer, M. Schäfer, A. Choudhary, N. Lal Sharma, R. Joos, C. Nawrath, J. H. Weber, W. Nie, *et al.*, High-fidelity distribution of triggered polarization-entangled telecom photons via a 36 km intra-city fiber network, Optica Quantum **2**, 274 (2024).
[9] C. Santori, D. Fattal, J. Vučković, G. S. Solomon, and Y. Yamamoto, Indistinguishable photons from a single-photon device, Nature **419**, 594 (2002).
[10] J. H. Weber, B. Kambs, J. Kettler, S. Kern, J. Maisch, H. Vural, M. Jetter, S. L. Portalupi, C. Becher, and P. Michler, Two-photon interference in the telecom c-band after frequency conversion of photons from remote quantum emitters, Nature nanotechnology **14**, 23 (2019).
[11] W. Nie, N. L. Sharma, C. Weigelt, R. Keil, J. Yang, F. Ding, C. Hopfmann, and O. G. Schmidt, Experimental optimization of the fiber coupling efficiency of gaas quantum dot-based photon sources, Applied Physics Letters **119** (2021).
[12] F. Basso Basset, F. Salusti, L. Schweickert, M. B. Rota, D. Tedeschi, S. F. Covre da Silva, E. Roccia, V. Zwiller, K. D. Jöns, A. Rastelli, and R. Trotta, Quantum teleportation with imperfect quantum dots, npj Quantum Information **7**, 7 (2021).
[13] T. van Leent, M. Bock, F. Fertig, R. Garthoff, S. Eppelt, Y. Zhou, P. Malik, M. Seubert, T. Bauer, W. Rosenfeld, W. Zhang, C. Becher, and H. Weinfurter, Entangling single atoms over 33 km telecom fibre, Nature **607**, 69 (2022).
[14] A. J. Hudson, R. M. Stevenson, A. J. Bennett, R. J. Young, C. A. Nicoll, P. Atkinson, K. Cooper, D. A. Ritchie, and A. J. Shields, Coherence of an entangled exciton-photon state, Phys. Rev. Lett. **99**, 266802 (2007).
